# Potential of sustainable, ecofriendly sterol derivatives as additives for water and oil repellency

**DOI:** 10.1038/s41598-026-47313-z

**Published:** 2026-04-03

**Authors:** Noraiza Suhaimi, Nurul Syahirah Shamsol Anuar, Masahiro Higashi, Yoshito Tanaka, Yu Mizote, Kanako Inoo, Yosuke Kishikawa, Kenshi Suzuki, Naoki Sunagawa, Yasuo Ohnishi, Takeaki Tezuka, Hirofumi Hara

**Affiliations:** 1 Department of Biotechnology, Graduate School of Agricultural and Life Sciences, The University of Tokyo, 1-1-1, Yayoi, Bunkyo-ku, Tokyo, 113- 8657 Japan; 2 Technology and Innovation Center, Daikin Industries, Ltd., 1-1, Nishi-Hitotsuya, Settsu, 566-8585 Osaka Japan; 3 Collaborative Research Institute for Innovative Microbiology (CRIIM), The University of Tokyo, 1-1-1, Yayoi, Bunkyo-ku, Tokyo, 113-8657 Japan; 4 Present Address: Department of Bioscience and Biotechnology, Graduate School of Bioresource and Bioenvironmental Sciences, Kyushu University, 744 Motooka Nishi-ku, Fukuoka, 819-0395 Japan

**Keywords:** Hydrophobicity, Oleophobicity, Isoprenoids, Sterol derivatives, Sustainable additives, Biochemistry, Chemistry, Materials science

## Abstract

An increasing global demand for sustainable packaging and protective materials has led to significant interest in developing effective water-resistant and oil-repellent materials from natural resources. This is because some of the current synthetic water-resistant and oil-repellent materials are made from organofluorine compounds. This study explored the potential of isoprenoid compounds, such as sterols, as biobased replacements for surface coating or packaging applications. The hydrophobicity and oleophobicity of various sterols and related structural compounds coated on silicon wafers and annealed at 60 °C and 140 °C were evaluated. As the annealing temperature increased, the hydrophobicity of sterol groups, such as cholesterol, ergosterol, β-sitosterol, and stigmasterol, increased, with their contact angles ranging from 100.7 ± 0.5° to 105.7 ± 1.1° when annealed at 140 °C. Moreover, cholesterol myristate and stearyl glycyrrhetinate exhibited good hydrophobicity and oleophobicity characteristics when annealed at 60 °C, with water contact angles of 106.4 ± 0.2° and 100.9 ± 0.7° and hexadecane contact angles of 46.1 ± 1.1° and 47.6 ± 5.0°, respectively. Based on the findings of this study, isoprenoids have potential as sustainable alternatives to water and oil repellents, particularly in applications where hydrophobicity is essential. Furthermore, this study offers valuable insights into the significant role of the annealing temperature in enhancing coating properties.

**Supplementary Information:**

The online version contains supplementary material available at 10.1038/s41598-026-47313-z.

## Introduction

Currently, the market for water and oil-repellent materials used in industrial applications is dominated by synthetic materials, primarily fluorinated compounds. These materials typically possess a rare combination of hydrophobic and oleophobic characteristics^1^. Materials with low surface energy properties are widely adopted across various sectors^2^, including food packaging, electronics, textiles, and paper products^1,3^, as they serve crucial functions in protecting contents, extending shelf life, and maintaining product integrity^4^. Efforts to improve environmental sustainability have led to intensive research on developing innovative biobased materials that exhibit water and oil repellency^5^.

Recent developments in bioresource utilization have opened promising avenues for creating sustainable alternatives. Among emerging candidates, isoprenoids are promising as natural amphiphiles, offering inherent water-repellent properties while maintaining biodegradability^6^. Thus, isoprenoids can be used as alternatives to food packaging materials. Isoprenoid compounds, such as sterols, are primarily naturally occurring hydrocarbons found in various organisms, and they can be developed as sustainable hydrophobic materials^7,8^. These compounds have a unique structure made up of repeating five-carbon isoprene units, which are crucial for imparting their predominantly hydrophobic character^9,10^.

Although some isoprenoids contain functional groups that introduce slight polarity, the presence of a nonpolar hydrocarbon backbone governs their overall water-repellent nature^11^. This hydrophobic trait is essential to diverse biological roles that isoprenoids play—from forming the structural core of cell membranes to acting as signaling molecules and contributing to the rich array of natural products found in plants and animals^12^. In addition to their inherent hydrophobic properties^13–15^ these compounds have various structures and functionalities that can be utilized for various applications, such as pharmaceuticals, cosmetics, and biofuels^16^. Furthermore, the presence of both hydrophobic and hydrophilic regions in some isoprenoids enables them to act as surfactants and emulsifiers^17,18^.

However, the hydrophobic interaction is weaker compared to fluorinated compounds, for example, Polytetrafluoroethylene (PTFE) or Perfluorooctane Sulfonate (PFOS), which possessed C-F bond that has a stronger attraction due to a larger dipole moment but also exhibit low polarizability^19^. This results in exceptionally low surface energy, allowing the material to exhibit both hydrophobic and oleophobic characteristics^20^. Even so, isoprenoid compounds with their biobased origin and biodegradability by specialized microbes^21–23^, may potentially offer more significant environmental benefits^24–26^ compared to the artificial compounds.

In this context, this study focuses on investigating the hydrophobicity and oleophobicity characteristics of selected isoprenoid compounds and assessing their potential as biobased materials, for example, for food wrapping or paper coating applications. An oleophobic surface repels oil molecules, while a hydrophobic surface repels water^27^. The number of chemical compounds that possess both of these characteristics, specifically chemical compounds with characteristics that match those of organofluorine compounds, is quite limited. Other than fluorinated compounds, Janus membrane is also a type of material that has both characteristics, however, with opposing properties to each other in a spongy form^28^. Thus, this research aims to leverage the natural abundance and renewability of isoprenoids with both properties to offer valuable insights for designing environmentally friendly, high-performance alternatives for various industrial applications.

## Results and Discussion

### Contact angle measurement of sterol

The hydrophobic and oleophobic properties of various sterols and related structural compounds were analyzed based on contact angle measurements against the coated silicon wafer annealed at different temperatures. Silicon wafer is used because it is widely known as a standard substrate for hydrophobicity and oleophobicity analysis, due to its precise wettability characteristics, making it easier to control the variability^29–31^. Since the aim is to investigate the potential of selected compounds for packaging application material, the contact angle of > 100° against a water droplet is considered good hydrophobicity, and a contact angle of > 40° against a hexadecane droplet is defined as moderate oleophobicity^32–35^. Annealing temperatures were chosen based on the fact that low temperatures (60 °C) enable controlled molecular reorganization, while high temperatures (140 °C) provide sufficient energy for major structural changes^36^. Additionally, comparing the hydrophobic and oleophobic properties between annealing temperatures of 60 °C and 140 °C enables the evaluation of the stability of the compounds and the homogeneity of the surface structure during the molding of wrapping papers or coating of materials. The data obtained from these measurements are summarized in Table 1 (actual images of contact angle measurements are provided in Supplemental Fig. 1).

**Table 1:** Contact angle measurements of coated sterol as well as cholesterol and its derivatives on a silicon wafer.

	Melting temperature (℃)	Hydrophobicity (against water drop)		*p*-value^*^	Oleophobicity (against hexadecane drop)		*p*-value^*^
		Annealing at 140℃	Annealing at 60℃		Annealing at 140℃	Annealing at 60℃	
*Negative and positive controls*							
Silicon wafer (no coating)	-	29.0 ± 1.5	31.2 ± 0.8		22.2 ± 2.5	20.2 ± 1.5	
Stearyl acrylate (monomer)	28	35.3 ± 2.2	33.8 ± 3.2		41.5 ± 6.2	28.3 ± 1.8	
Stearyl acrylate (polymer)	-	38.2 ± 1.7	109.7 ± 0.3	0.000	31.8 ± 0.3	41.8 ± 0.1	0.000
*Sterol*,* cholesterol*,* and it’s derivatives*							
Cholesterol	148–151	100.7 ± 0.5	93.8 ± 1.0	0.002	30.6 ± 1.4	26.1 ± 0.6	0.016
β-sitosterol	140–146	104.3 ± 0.2	93.1 ± 1.6	0.005	28.8 ± 3.7	31.1 ± 1.4	0.403
Ergosterol	156–162	101.1 ± 1.9	96.7 ± 2.0	0.052	30.0 ± 2.9	33.2 ± 3.0	0.262
Stigmasterol	166–170	105.7 ± 1.1	97.6 ± 2.7	0.023	24.9 ± 3.1	22.3 ± 1.0	0.688
Cholesterol benzoate	147–152	80.3 ± 5.0	100.5 ± 1.4	0.014	22.4 ± 5.0	24.3 ± 4.9	0.642
Cholesterol butyrate	95–97	103.6 ± 1.9	100.4 ± 0.5	0.096	26.9 ± 2.2	28.2 ± 4.7	0.702
Cholesterol decanoate	81–84	91.8 ± 0.6	100.8 ± 1.1	0.001	28.5 ± 3.0	27.4 ± 2.0	0.640
Cholesterol myristate	84	105.5 ± 7.5	106.4 ± 0.2	0.854	35.8 ± 1.4	46.1 ± 1.1	0.001
Stearyl glycyrrhetinate	75–79	58.1 ± 8.7	100.9 ± 0.7	0.013	28.8 ± 5.5	47.6 ± 5.0	0.012

*Statistical analysis between the values at 140℃ and 60℃ of each compound was conducted by Student’s *t*-test.

As shown in Table 1, the contact angles of the stearyl acrylic polymer (positive control) when annealed at 60 °C were 109.7 ± 0.3° (water) and 41.8 ± 0.1° (hexadecane), indicating reliable hydrophobic and oleophobic characteristics, respectively. This is a significant improvement compared to the contact angles measured for the polymer when annealed at 140 °C. Relative to the positive control, cholesterol showed sufficient hydrophobicity and oleophobicity, with contact angles of 100.7 ± 0.5° and 30.6 ± 1.4° when annealed at 140 °C, respectively. The rigid structure of the ring and flexible hydrocarbon tail may contribute to the hydrophobic characteristic^37^(Table 2). However, contact angles for water and hexadecane droplets significantly decreased to 93.8 ± 1.0° and 26.1 ± 0.6°, respectively, when annealed at 60 °C. Owing to these variations, other sterol compounds were also investigated.

**Table 2:** Structural comparison of the tested sterol, as well as cholesterol and its derivatives.

Compounds	Chemical structure	Key structural feature	Key structural order	Hydrophobic/oleophobic performance
Cholesterol		Alkyl side chain located at C17 of the D-ring	Rigid ring structure	As parent structure
β-sitosterol		Ethyl group addition to the alkyl side chain	High crystallinity order with longer carbon side chain	Less efficient compared to cholesterol
Ergosterol		Methyl group addition + double bond to the alkyl side chain	Tight molecular packing	More hydrophobic than cholesterol
Stigmasterol		Ethyl group addition + double bond to the alkyl side chain	Able to self-assemble to form compact crystalline order	More hydrophobic than cholesterol
Cholesterol benzoate		Benzoate group to the C3 of A-ring	Condense molecular order	Less hydrophobic than cholesterol
Cholesterol butylate		C4 fatty acid chain to the C3 of the A-ring	Highly rigid and packed molecular order	More hydrophobic than cholesterol
Cholesterol decanoate		C10 fatty acid chain to the C3 of the A-ring	Highly rigid and packed molecular order	More hydrophobic than cholesterol
Cholesterol myristate		C14 fatty acid chain to the C3 of the A-ring	Highly ordered & reversible smectic and solid phases	Excellent hydrophobicity and oleophobicity
Stearyl glycyrrhetinate		Glycyrrhetinic acid + C18 alkyl chain	Bulky and packed molecular order	Better hydrophobicity and oleophobicity at 60℃

Ergosterol (with a double bond and an additional methyl group at the side chain), β-sitosterol (with an extra ethyl group at the side chain), and stigmasterol (with a double bond and an additional ethyl group at the side chain) were tested. These compounds were chosen because they enable the analysis of the effect of an additional methyl or ethyl group and a double bond on the side chain on the contact angle. These compounds exhibited higher water contact angles of 101.1 ± 1.9°, 104.3 ± 0.2°, and 105.7 ± 1.1° when annealed at 140 °C. These values were better than the result of cholesterol (100.7 ± 0.5°). Their hydrophobic characteristics lie in their key structural order; ergosterol, is known to have tight molecular packing^38^, β-sitosterol has high crystalline order^39^, and stigmasterol can self-assemble and form compact crystalline order^40^. However, when these compounds were annealed at 60 °C, the contact angles decreased to 96.7 ± 2.0°, 93.1 ± 1.6°, and 97.6 ± 2.7°.

On the other hand, no specific trend was observed in the hexadecane contact angles of these sterol compounds. Changes in annealing temperature did not significantly affect the contact angle of hexadecane against these compounds, as shown in Table 1. For ergosterol and β-sitosterol, albeit insignificantly, the contact angles of the hexadecane droplet increased from 30.0 ± 2.9° to 33.2 ± 3.0° and from 28.8 ± 3.7° to 31.1 ± 1.4°, respectively, when the annealing temperature decreased from 140 °C to 60 °C.

Statistical analysis, as shown in Supplementary Table S1, confirmed that the contact angles of β-sitosterol and stigmasterol annealed at 140 °C were statistically significant compared to that of cholesterol (*p* < 0.05), indicating an improvement in surface hydrophobicity. However, the comparison between cholesterol and ergosterol was not statistically significant, which can be attributed to a large variance in the ergosterol dataset. Based on these results, it is safe to suggest that the addition of an ethyl chain to the cholesterol by structure, as observed on β-sitosterol and stigmasterol, improves hydrophobicity^41,42^.

To support these findings, laser microscopy analysis was performed to examine the uniformity of sterol compound films on the silicon wafer and subsequently analyze the efficacy of coating at an annealing temperature of 140 °C, as all compounds showed good hydrophobicity (contact angles ≥ 100°) at this temperature. Supplemental Fig. 2 and Supplemental Fig. 3 illustrate the laser microscopy images of cholesterol, ergosterol, β-sitosterol, and stigmasterol films on a silicon wafer. These images (left panels) facilitate the qualitative visualization of the surface morphology, where bright spots indicate surface irregularities or particulate features. The corresponding 2D surface-height maps (right panels) represent the topographical distribution of coating. Furthermore, warm colors (red/yellow) denote elevated regions, and cool colors (blue/green) indicate lower areas.

Supplemental Fig. 2 reveals that the cholesterol and β-sitosterol films formed a uniform coating on the silicon wafer. The figures also show images of the laser observation and surface height of cholesterol and β-sitosterol, where the surface appears smooth. This indicates their good spreading, especially when magnified at 20×. These results are likely related to the heat response and crystallization tendencies of sterol compounds during annealing^43^.

Annealing is a treatment in which a coated material is heated at a certain temperature to improve its adhesion, remove solvents or unwanted residues, and enhance its structural or functional properties^44^. An optimal annealing temperature enhances molecular mobility and promotes film spreading^45,46^. Because cholesterol and β-sitosterol have a similar melting point range of 140–151 °C (Table 1), uniform coating formation strongly depends on the optimum annealing temperature relative to the melting point of sterols. This uniformity is directly linked to improved hydrophobic properties, as surface defects or aggregates disrupt water repellency^46,47^.

Contrary to the cholesterol and β-sitosterol films, neither ergosterol nor stigmasterol formed a perfectly uniform coating. In the laser microscopy results (Supplemental Fig. 3), a cloudy appearance is shown in both the optical view and surface height images. This indicates microscale inhomogeneity rather than smoothness. Previous reports suggested that the melting points of ergosterol and stigmasterol ranged from 156 °C to 170 °C (Table 1), thereby supporting the hypothesis that the selection of an optimum annealing temperature is intrinsically linked to the melting point of each compound.

Based on these observations, it is safe to suggest that, while addition of alkyl chain helps to enhance hydrophobicity, the homogeneity of coating also plays an important role in ensuring a reliable hydrophobicity reading. Therefore, to combine both factors, only β-sitosterol showed a significant hydrophobic characteristic when compared to cholesterol.

### Effects of alkyl chains on the contact angles of cholesterol derivatives

With reference to the above-mentioned results, several cholesterol derivatives with alkyl chain attachments were investigated. The selected compounds were cholesterol benzoate, cholesterol butyrate, cholesterol decanoate, cholesterol myristate, and stearyl glycyrrhetinate (a compound with a related structure). These compounds are known to have highly rigid molecular order due to the alkyl chain attachments, and thus, they may exhibit better hydrophobic characteristics^48,49^. Cholesterol benzoate has a benzoate group that forms an ester bond with the hydroxyl group (-OH) located at carbon number 3 of cholesterol. At an annealing temperature of 140 °C, this compound exhibited weak hydrophobicity and no oleophobic characteristics. However, these characteristics improved when it was annealed at 60 °C, with contact angles improving from 80.3 ± 5.0° and 22.4 ± 5.0° to 100.5 ± 1.4° and 24.3 ± 4.9°, respectively (Table 1).

Notably, cholesterol butyrate, cholesterol decanoate, and cholesterol myristate share a common core structural feature in which the -OH at the carbon number 3 position of cholesterol is esterified to a fatty acid chain with differing lengths: butyrate (C4), decanoate (C10), and myristate (C14). Hence, an increase in the water contact angle was expected with an increase in the number of alkyl chains. Instead, contact angles were inconsistent when they were annealed at 140 °C. Cholesterol decanoate exhibited weaker hydrophobicity (91.8 ± 0.6°), while the contact angles of cholesterol butyrate and myristate were 103.6 ± 1.9° and 105.5 ± 7.5°, respectively. This may be due to cholesterol decanoate experiencing conformational entropy at high temperatures^49,50^. Interestingly, only cholesterol myristate exhibited excellent oleophobic characteristics at both annealing temperatures, with the hexadecane contact angles of 35.8 ± 1.4° (140 °C) and 46.1 ± 1.1° (60 °C). This may be due to the ability of cholesterol myristate to have reversible smectic and solid phase properties, which contribute to these findings^51^.

On the other hand, the hexadecane contact angles of the other three compounds were below 30° and no significant changes were observed between the two annealing temperatures, 140 °C and 60 °C (Table 1; Supplementary Table S2).

Additionally, significant hydrophobicity and oleophobicity characteristics were observed for the stearyl glycyrrhetinate coating. Because its parent structure is glycyrrhetinic acid, this compound is not classified as a cholesterol derivative. Nevertheless, it was included in this study because it exhibits structural and functional similarities to ester-type derivatives of bioactive molecules. As shown in Table 1, a reduction in the annealing temperature significantly increased the water droplet contact angle against stearyl glycyrrhetinate from 58.1 ± 8.7° to 100.9 ± 0.7° (Supplementary Table S1). Most importantly, this substantial temperature reduction significantly increased the contact angle against the hexadecane droplet, reaching 47.6 ± 5.0° from 28.8 ± 5.5° (Supplementary Table S2). This implies an enhancement in the oleophobicity characteristics, as needed.

Similar to the results presented above, the observed variations in contact angles with different annealing temperatures highlight the importance of optimizing the conditions for each compound. Sterol groups have higher melting points; therefore, the annealing temperature of 140 °C potentially helps create a more uniform coating, subsequently contributing to a higher water contact angle. By contrast, stearyl glycyrrhetinate and cholesterol myristate have lower melting points (~ 75–84 °C). Consequently, annealing at a lower temperature (60 °C) is more effective for stearyl glycyrrhetinate and produces a pronounced change in the oleophobicity of cholesterol myristate (Supplementary Table S2). The contrasting results at 140 °C and 60 °C may occur when a compound undergoes thermal degradation or structural alteration at elevated temperatures^52,53^. However, this has yet to be confirmed with Thermogravimetric Analysis (TGA), Differential Scanning Calorimetry (DSC), or chemical analysis.

Compared with the water contact angle of cholesterol (Supplementary Table S1), those of cholesterol derivatives and stearyl glycyrrhetinate significantly differed (*p* < 0.05) at 60 °C. In addition, cholesterol myristate showed consistent and significant oleophobicity values at both temperatures. Meanwhile, the hydrophobic results for cholesterol butyrate and cholesterol myristate at 140 °C, despite showing a higher contact angle, did not reveal a significant difference (*p* > 0.05). This is because of a high variability in their measurements, as indicated by the large standard deviation value (Supplementary Table S1). Based on these findings, it can be concluded that attachment of C14 to the parent compound (cholesterol) improves the hydrophobicity and oleophobicity characteristics at an annealing temperature of 60 °C, despite the variability^54^.

### Potential of sterols, cholesterol derivatives, and stearyl glycyrrhetinate as alternative materials from bioresources

The sterol group and cholesterol derivatives demonstrated promising water and oil repellency at a suitable annealing temperature, indicating their potential as alternative biobased materials to organofluorine compounds. As mentioned above, the contact angles of the stearyl acrylate polymer (positive control) were 109.7 ± 0.3° and 41.8 ± 0.1°, while those of the stearyl acrylate monomer were 33.8 ± 3.2° and 28.3 ± 1.8° for water and hexadecane at 60 °C, respectively. Using these values as a reference and comparing the results exhibited by compounds listed in Table 1 indicate that sterols and cholesterol derivatives have potential as hydrophobic agents, although further optimization is needed to achieve comparable oleophobicity.

Notably, these compounds demonstrated high contact angles in the monomeric form, in contrast to the results obtained for stearyl acrylate. The ability of organic compounds to display high contact angles on silicon wafer substrates in their monomeric form rather than as polymers offers distinct advantages in surface modification for hydrophobicity and oleophobicity characterizations because this promotes a more organized molecular orientation^55^ and less aggregation when in contact with solvents^56^. This enables fine-tuned control over surface wettability^57^.

Utilizing these monomers in polymerization or copolymerization strategies, the hydrophobic and oleophobic characteristics of them can be further enhanced, and a range of polymers can be developed for which surface interactions can be fine-tuned^58^. For instance, incorporating hydrophobic comonomers can amplify the water-repellent nature of the resulting polymers, while the strategic addition of hydrophilic comonomers can introduce a balance of hydrophilic and hydrophobic domains, yielding amphiphilic materials with unique self-assembly behaviors. This ability to manipulate surface properties through polymerization expands the potential applications of isoprenoids in various fields, especially as alternatives to organofluorine compounds. However, there is also a need for the comprehensive testing of these alternatives, including testing their persistence, bioaccumulation potential, and toxicity to humans and the environment.

### Future perspectives and potential applications

The studied compounds, if explored and developed carefully as mentioned in the previous section, could be a versatile building block for sustainable next-generation functional materials. Sterols and their derivatives were already found to possess anti-fouling activities^59,60^. Subsequently, if superhydrophobic characteristics could be developed with the copolymerization technique, it could potentially graft both anti-fouling and self-cleaning bio-coating materials^61,62^, that prevent bacterial adhesion by entropic repulsion, without the use of harmful biocides.

Furthermore, the integration of these compounds with other biobased materials to form biobased packaging films shows promise in addressing the permeability issues of sustainable plastics, providing a reliable water-vapor barrier^63^. As material science moves toward green chemistry, the biodegradability and structural rigidity of sterol compounds may provide a viable substitute for artificial fluorocarbons in the development of superhydrophobic and self-healing interfaces.

### Conclusion

This study reveals that even though more optimizations are needed, sterol groups, cholesterol myristate, and stearyl glycyrrhetinate offer significant advantages over current synthetic materials in terms of environmental impact and biocompatibility, making them promising for use as sustainable alternatives. Although some compounds approached the hydrophobicity of stearyl acrylic polymer, further research is needed to optimize their surface properties and potentially surpass the performance of stearyl acrylate in terms of oleophobicity.

## Methods

### Chemicals

Stearyl acrylate was used as a positive control. Several sterols and related structural compounds available in the market were selected based on their distinguished chemical structures and their ability to have both hydrophobic and oleophobic characteristics. All sterol compounds (β-sitosterol, cholesterol, ergosterol, and stigmasterol), several cholesterol derivative compounds (such as cholesterol benzoate, cholesterol butyrate, cholesterol decanoate, and cholesterol myristate), and stearyl glycyrrhetinate were purchased from Tokyo Chemical Industry (TCI), Japan.

### Positive control preparation

Stearyl acrylate was used in both monomer and polymer form as positive controls. To synthesize the corresponding polymer, 2 g stearyl acrylate was dissolved in 8 g toluene, followed by the addition of 38 mg 2,2’-azobisisobutyronitrile (AIBN). The mixture was heated and stirred at 75 °C for 24 h under a nitrogen atmosphere. The reaction mixture was then purified by reprecipitation with acetone and dried under reduced pressure to obtain the desired stearyl acrylic polymer. Molecular weight (GPC): Mw 18.8 K, Mn 9.9 K, Purity (^1^H-NMR, CDCl_3_): Residual stearyl acrylate content < 1%.

### Substrate preparation and coating on silicon wafers

All chemical compounds were prepared and dissolved in chloroform, with a weight/weight concentration of 1%. A silicon wafer (2–960-15; AS ONE Corporation, Japan) was used as the substrate for contact angle measurements. The prepared silicon wafer had a size of 30 mm × 30 mm and was washed with acetone for 30 min ultrasonically. The as-prepared silicon substrates were then dried and kept in a clean glass container. The prepared chemical solution in chloroform was applied to the silicon substrate using a spin coater (Opticoat MS-B150, MIKASA) at 2000 rpm for 25 s. A total of 500 µL of the chemical solution was deposited onto the surface of the silicon substrate until it completely covered the surface. The as-coated silicon substrate was then incubated in a preheated oven at annealing temperatures of 140 °C and 60 °C for 1 min. The choice of an annealing temperature primarily depends on the melting point of analyzed compounds to avoid their decomposition or degradation and achieve a proper molecular rearrangement^64^.

### Contact angle measurement

The contact angles of water and hexadecane droplets were measured using a contact angle meter (Drop Master DM-701, Kyowa Interface Science Co., Ltd). A total of 2 µL of water or hexadecane was dropped onto the coated silicon substrate. All measurements were triplicated.

### Laser microscopy observation

Surface roughness parameters—Sa (arithmetical mean height) and Sz (maximum height)—were obtained for each sample using a 3D laser scanning microscope (VK-X3000 Series; Keyence, Tokyo, Japan). These two parameters were analyzed at ×5 and ×20 objective and laser light (intensity) over areas of 10 mm × 0.6 mm and 2,500 μm × 1,500 μm, respectively. Three points of measurement were selected on the silicon wafer coated with each substrate.

### Statistical analysis

All statistical analyses were conducted using unpaired two-tailed Student’s *t*-tests, with significance set at *p* < 0.05.

## Supplementary Information

Below is the link to the electronic supplementary material.

Supplementary Material 1

## Data Availability

All data generated and analyzed in this study are included in this article.

## References

[1] Ahmad, D., van den Boogaert, I., Miller, J., Presswell, R. & Jouhara, H. Hydrophilic and hydrophobic materials and their applications. *Energy Sources, Part A: Recovery, Utilization, and Environmental Effects* **40**, 2686–2725 (2018).

[2] Tang, W., Huang, Y. & Qing, F.-L. Synthesis and characterization of fluorinated polyacrylate graft copolymers capable as water and oil repellent finishing agents. *J. Appl. Polym. Sci.* **119**, 84–92 (2011).

[3] Omerogullari Basyigit, Z. & Coskun, H. Enhancing antibacterial and water-repellent properties for the production of high-performance fabrics in home textiles. *Fibers Polym.* **25**, 1789–1804 (2024).

[4] Li, Y. et al. Synthesis and Performance Analysis of Green Water and Oil-Repellent Finishing Agent with Di-Short Fluorocarbon Chain. *Molecules*10.3390/molecules28083369 (2023). 37110600 10.3390/molecules28083369 PMC10144625

[5] Rungruangkitkrai, N. et al. Water Repellent Coating in Textile, Paper and Bioplastic Polymers: A Comprehensive Review. *Polymers (Basel).*10.3390/polym16192790 (2024). 39408499 10.3390/polym16192790 PMC11479018

[6] Sheibani, S. et al. Sustainable strategies for using natural extracts in smart food packaging. *Int. J. Biol. Macromol.* **267**, 131537 (2024). 38608975 10.1016/j.ijbiomac.2024.131537

[7] Shapira, A., Berman, P., Futoran, K., Guberman, O. & Meiri, D. Tandem mass spectrometric quantification of 93 terpenoids in Cannabis using static headspace injections. *Anal. Chem.* **91**, 11425–11432 (2019). 31369251 10.1021/acs.analchem.9b02844

[8] Pizzo, J. S. et al. Quantifying terpenes in tomato leaf extracts from different species using gas chromatography-mass spectrometry (GC-MS). *Anal. Biochem.* **689**, 115503 (2024). 38453049 10.1016/j.ab.2024.115503

[9] Azekriti, S., Mehenaoui, K., Ehrenfeld, F., Laffore, A. & Save, M. Amphiphilic poly(β-myrcene-co-acrylic acid) copolymers synthesized by nitroxide-mediated copolymerization as stabilizers of terpene-based waterborne latex. *Biomacromolecules* **26**, 1111–1127 (2025). 39879075 10.1021/acs.biomac.4c01444

[10] Chappell, J. The Biochemistry and Molecular Biology of Isoprenoid Metabolism. *Plant. Physiol.* **107**, 1–6 (1995). 12228337 10.1104/pp.107.1.1 PMC161157

[11] Metwarebio & Metwarebio Sterol Lipids: Structure, Function, and Their Role in Health and Disease. https://www.metwarebio.com/sterol-lipids-structure-function-health-disease/ (2024).

[12] Tarkowská, D. & Strnad, M. Isoprenoid-derived plant signaling molecules: Biosynthesis and biological importance. *Planta* **247**, 1051–1066 (2018). 29532163 10.1007/s00425-018-2878-x

[13] Martins, M. A. R. et al. Greener terpene–terpene eutectic mixtures as hydrophobic solvents. *ACS Sustainable Chem. Eng.* **7**, 17414–17423 (2019).

[14] Schaeffer, N., Martins, M. A. R., Neves, C. M. S. S., Pinho, S. P. & Coutinho, J. A. P. Sustainable hydrophobic terpene-based eutectic solvents for the extraction and separation of metals. *Chem. Commun.* **54**, 8104–8107 (2018). 10.1039/c8cc04152k 29972155

[15] Pichersky, E. & Raguso, R. A. Why do plants produce so many terpenoid compounds?. *New Phytol.* **220**, 692–702 (2018). 27604856 10.1111/nph.14178

[16] Tetali, S. D. Terpenes and isoprenoids: A wealth of compounds for global use. *Planta* **249**, 1–8 (2019). 30467631 10.1007/s00425-018-3056-x

[17] Kato, T. et al. Micelle structures in aqueous solutions of glucose-based surfactants having an isoprenoid-type hydrophobic chain. *J. Colloid Interface Sci.* **312**, 122–129 (2007). 17547933 10.1016/j.jcis.2006.09.030

[18] Eskandar, N. G., Simovic, S. & Prestidge, C. A. Chemical stability and phase distribution of all-trans-retinol in nanoparticle-coated emulsions. *Int. J. Pharm.* **376**, 186–194 (2009). 19422895 10.1016/j.ijpharm.2009.04.036

[19] Gong, L. et al. Unraveling the hydrophobic interaction mechanisms of hydrocarbon and fluorinated surfaces. *J. Colloid Interface Sci.* **635**, 273–283 (2023). 36587579 10.1016/j.jcis.2022.12.084

[20] Sagisaka, M., Darmanin, T., Guittard, F. & Eastoe, J. New fluorine-free low surface energy surfactants and surfaces. *J. Colloid Interface Sci.* **690**, 137229 (2025). 40112528 10.1016/j.jcis.2025.03.018

[21] Nkem, B. M. et al. Isolation, identification and diesel-oil biodegradation capacities of indigenous hydrocarbon-degrading strains of *Cellulosimicrobium cellulans* and *Acinetobacter baumannii* from tarball at Terengganu beach, Malaysia. *Mar. Pollut. Bull.* **107**, 261–268 (2016). 27085593 10.1016/j.marpolbul.2016.03.060

[22] Srivastva, N., Singh, A., Bhardwaj, Y. & Dubey, S. K. Biotechnological potential for degradation of isoprene: A review. *Crit. Rev. Biotechnol.* **38**, 587–599 (2018). 29233013 10.1080/07388551.2017.1379467

[23] Song, S. et al. Knockout of glycerol metabolic pathways enables efficient mycolicibacterial phytosterol conversion using glycerol as cosovlent. *Appl. Microbiol. Biotechnol.* **109**, 169 (2025). 40690050 10.1007/s00253-024-13360-7 PMC12279570

[24] Gururani, P. et al. Bio-based food packaging materials: A sustainable and holistic approach for cleaner environment- a review. *Current Research in Green and Sustainable Chemistry* **7**, 100384 (2023).

[25] Oliveira, I. et al. Sustainability in Bio-Based Edible Films, Coatings, and Packaging for Small Fruits. *Applied Sciences* **15**, 1462 (2025).

[26] Ncube, L. K., Ude, A. U., Ogunmuyiwa, E. N., Zulkifli, R. & Beas, I. N. Environmental Impact of Food Packaging Materials: A Review of Contemporary Development from Conventional Plastics to Polylactic Acid Based Materials. *Materials*10.3390/ma13214994 (2020). 33171895 10.3390/ma13214994 PMC7664184

[27] Drelich, J., Chibowski, E., Meng, D. D. & Terpilowski, K. Hydrophilic and superhydrophilic surfaces and materials. *Soft Matter* **7**, 9804–9828 (2011).

[28] Kuang, X. et al. Biomimetic Janus membrane with spongy channels for directional liquid transport. *Nat. Commun.* **16**, 10001 (2025). 41238539 10.1038/s41467-025-64964-0 PMC12618621

[29] Yang, X. M., Zhong, Z. W., Diallo, E. M., Wang, Z. H. & Yue, W. S. Silicon wafer wettability and aging behaviors: Impact on gold thin-film morphology. *Mater. Sci. Semicond. Process.* **26**, 25–32 (2014).

[30] Chang, Z. et al. Hydrophobic nanostructures fabricated by ferric nitrate etching method on single crystalline silicon surface. *Colloids Surf. A Physicochem. Eng. Asp.* **583**, 123999 (2019).

[31] WaferPro. What is a silicon wafer? What is it used for? WaferPro https://waferpro.com/what-is-a-silicon-wafer/ (2024).

[32] Song, Y., Dunleavy, M. & Li, L. How to make plastic surfaces simultaneously hydrophilic/oleophobic?. *ACS Appl. Mater. Interfaces* **15**, 31092–31099 (2023). 37326374 10.1021/acsami.3c06787 PMC10316401

[33] Kessman, A. & Cairns, D. Hydrophobic and oleophobic coatings. US Patent, US20110250422A1 (2018).

[34] Jhaveri, J. H. & Murthy, Z. V. P. A comprehensive review on anti-fouling nanocomposite membranes for pressure driven membrane separation processes. *Desalination* **379**, 137–154 (2016).

[35] Amoresano, A., Nebbioso, V., Roscioli, S. & Aronne, A. Characterization of hydrophobic surfaces by experimental study of static and dynamic contact angles. *J. Phys. Conf. Ser.* **3143**, 012026 (2025).

[36] Barriuso, B., Otaegui-Arrazola, A., Menéndez-Carreño, M., Astiasarán, I. & Ansorena, D. Sterols heating: Degradation and formation of their ring-structure polar oxidation products. *Food Chem.* **135**, 706–712 (2012). 22868149 10.1016/j.foodchem.2012.05.027

[37] Shepelenko, M. et al. Polymorphism, structure, and nucleation of Cholesterol·H_2_O at aqueous interfaces and in pathological media: Revisited from a computational perspective. *J. Am. Chem. Soc* **144**, 5304–5314 (2022). 35293741 10.1021/jacs.1c10563 PMC8972249

[38] Ermakova, E. & Zuev, Y. Effect of ergosterol on the fungal membrane properties. All-atom and coarse-grained molecular dynamics study. *Chem. Phys. Lipids*. **209**, 45–53 (2017). 29122611 10.1016/j.chemphyslip.2017.11.006

[39] Christiansen, L. I., Rantanen, J. T., Von Bonsdorff, A. K., Karjalainen, M. A. & Yliruusi, J. K. A novel method of producing a microcrystalline β-sitosterol suspension in oil. *Eur. J. Pharm. Sci.* **15**, 261–269 (2002). 11923058 10.1016/s0928-0987(01)00223-8

[40] Bag, B. G. & Barai, A. C. Self-assembly of naturally occurring stigmasterol in liquids yielding a fibrillar network and gel. *RSC Adv.* **10**, 4755–4762 (2020). 35495245 10.1039/c9ra10376g PMC9049162

[41] Verma, C., Quraishi, M. A. & Rhee, K. Y. Hydrophilicity and hydrophobicity consideration of organic surfactant compounds: Effect of alkyl chain length on corrosion protection. *Adv. Colloid Interface Sci.* **306**, 102723 (2022). 35816779 10.1016/j.cis.2022.102723

[42] Widati, A. A., Fahmi, M. Z., Sakti, S. C. W., Budiastanti, T. A. & Purbaningtias, T. E. Hydrophobic Material: Effect of Alkyl Chain Length on the Surface Roughness. *Journal of Manufacturing and Materials Processing*10.3390/jmmp6050110 (2022).

[43] Li, H. et al. Influence of annealing temperature on the structure and properties of Ti3AlC2 coatings by FCVA method. *Mater. Charact.* **194**, 112421 (2022).

[44] Shivaraj, B. W., Murthy, H. N. N., Krishna, M. & Satyanarayana, B. S. Effect of annealing temperature on structural and optical properties of dip and spin coated ZnO thin films. *Procedia Materials Science* **10**, 292–300 (2015).

[45] Guo, D., Ikeda, S., Saiki, K., Miyazoe, H. & Terashima, K. Effect of annealing on the mobility and morphology of thermally activated pentacene thin film transistors. *J. Appl. Phys.* **99**, 094502 (2006).

[46] Bougharouat, A., Touka, N., Talbi, D. & Baddari, K. Hydrophobic properties of CuO thin films obtained by sol-gel spin coating technique-annealing temperature effect. *Ann. Chim. Sci. Mater.* **45**, 439–445 (2021).

[47] Eftekhari, L. & Ghasemi, M. Analysing the surface morphology of annealed FTO/ZnS bilayer films: Stereometric, fractal, and wettability approaches. *Sci. Rep.* **14**, 14262 (2024). 38902309 10.1038/s41598-024-65118-w PMC11190240

[48] Hoppe, C. E. & Williams, R. J. J. Tailoring the self-assembly of linear alkyl chains for the design of advanced materials with technological applications. *J. Colloid Interface Sci.* **513**, 911–922 (2018). 29079386 10.1016/j.jcis.2017.10.048

[49] Tan, Y. et al. Crystalline alkyl side chain enhanced hydrophobicity–oleophobicity for transparent and durable low-surface-energy coatings based on short fluorinated copolymers. *Macromolecules* **57**, 5189–5199 (2024).

[50] Small, D. M. Crystal structure of cholesteryl decanoate. *J. Lipid Res.* **20**, 753–759 (1979). 490051

[51] Churchill, D. & Bailey, L. W. Surface tension of cholesteric liquid crystals: Cholesteryl myristate. *Mol. Cryst.* **7**, 285–293 (1969).

[52] Yu, Y. et al. Effect of Ultra-High Temperature Degradation on the Physical Properties and Chemical Structure of an AMPS-Based Copolymer Oil-Well Cement Additive PADIM in Aqueous Solution. *Polymers*10.3390/polym17050591 (2025). 40076084 10.3390/polym17050591 PMC11902507

[53] Moreira, K. C. C. S. R., Dalmaschio, C. J., Soares, E. J. & Nascimento, A. Thermal Degradation and Rheological Behavior of Xanthan Gum: Kinetics, Mechanism, and Aging Effects.. *ChemistrySelect* **10**, e00855 (2025).

[54] Li, H.-L. et al. Alkyl side-chain engineering for enhanced anti-wettability and stability in non-fluorinated liquid-repellent coatings. *ACS Appl. Mater. Interfaces* **17**, 48863–48872 (2025). 40825099 10.1021/acsami.5c08629

[55] Yang, J., Yim, S. & Jones, T. S. Molecular-orientation-induced rapid roughening and morphology transition in organic semiconductor thin-film growth. *Sci. Rep.* **5**, 9441 (2015). 25801646 10.1038/srep09441 PMC4371086

[56] Cimatu, K. L. A. et al. Polymer-solvent interaction and conformational changes at a molecular level: Implication to solvent-assisted deformation and aggregation at the polymer surface. *J. Colloid Interface Sci.* **616**, 221–233 (2022). 35203035 10.1016/j.jcis.2022.02.006

[57] Guo, R. et al. Exploiting molecular dynamics in composite coatings to design robust super-repellent surfaces. *Adv. Sci.* **9**, 2104331 (2022). 10.1002/advs.202104331 PMC8867138 34997692

[58] Hukum, K. O., Caliskan, T. D., Caykara, T. & Demirel, G. Toward water and oil repellent coating: Synthesis of fluorinated methacrylate-glycidyl methacrylate copolymers. *ACS Omega* **9**, 34650–34660 (2024). 39157152 10.1021/acsomega.4c03275 PMC11325509

[59] Nik Mohd Sukrri, N. N. A. et al. Antifouling activity of Malaysian green seaweed *Ulva lactuca* and its isolated non-polar compound. *Heliyon* **10**, e38366 (2024). 39397965 10.1016/j.heliyon.2024.e38366 PMC11467595

[60] Elkady, E. M., Tadros, H. R. Z., Soliman, Y. A., Raafat, M. & Abdel-Tawab, A. M. Marine antifouling agents based on bioactive compounds isolated from Red Sea soft corals *Sarcophyton glaucum* and *Sclerophytum leptoclados*. *Egypt. J. Aquat. Res.* **50**, 234–240 (2024).

[61] Wang, J., Li, L., Wu, Y. & Liu, Y. Design and Application of Antifouling Bio-Coatings.. *Polymers (Basel).*10.3390/polym17060793 (2025). 40292673 10.3390/polym17060793 PMC11945268

[62] Zhang, B., Xue, X., Zhao, L. & Hou, B. Transparent Superhydrophobic and Self-Cleaning Coating.. *Polymers (Basel).*10.3390/polym16131876 (2024). 39000731 10.3390/polym16131876 PMC11244105

[63] Bora, N. S., Kalita, P., Mary, S. E. & Roy, S. Lipid-based food packaging film/coating in postharvest fruit preservation. *Food Packag. Shelf Life* **50**, 101569 (2025).

[64] Choi, J. et al. Annealing temperature effect on the surface properties of the MoSe thin films. *physica status solidi (a)* **220**, 2300477 (2023).

