# Supplementary material for: Potential of sustainable, ecofriendly sterol derivatives as additives for water and oil repellency
Source: Sci Rep. 2026 Apr 3;16:15979. doi: 10.1038/s41598-026-47313-z (PMC13194897; doi:10.1038/s41598-026-47313-z)
Supplement: Supplementary file 1 — Supplementary Material 1 [file 41598_2026_47313_MOESM1_ESM.docx]

**Table S1.** Contact angle measurements of water droplets and statistical comparisons of cholesterol derivatives and stearyl glycyrrhetinate with cholesterol at different annealing temperatures

|  | Contact angle* | | *p*-value** | |
| --- | --- | --- | --- | --- |
|  | Annealing at 140℃ | Annealing at 60℃ | Annealing at 140℃ | Annealing at 60℃ |
| Cholesterol | 100.7±0.5 | 93.8±1.0 |  |  |
| β-sitosterol | 104.3±0.2 | 93.1±1.6 | 0.003 | 0.552 |
| Ergosterol | 101.1±1.9 | 96.7±2.0 | 0.776 | 0.101 |
| Stigmasterol | 105.7±1.1 | 97.6±2.7 | 0.006 | 0.118 |
| Cholesterol benzoate | 80.3±5.0 | 100.5±1.4 | 0.019 | 0.004 |
| Cholesterol butyrate | 103.6±1.9 | 100.4±0.5 | 0.112 | 0.002 |
| Cholesterol decanoate | 91.8±0.6 | 100.8±1.1 | 3.95x10^-5^ | 0.001 |
| Cholesterol myristate | 105.5±7.5 | 106.4±0.2 | 0.378 | 0.001 |
| Stearyl glycyrrhetinate | 58.1±8.7 | 100.9±0.7 | 0.014 | 0.001 |

*Each measurement was repeated three times (n = 3).

**The *p*‑value represents the significance of the difference compared with the contact angle of cholesterol.

**Table S2.** Contact angle measurements of hexadecane droplets and statistical comparisons of cholesterol derivatives and stearyl glycyrrhetinate with cholesterol at different annealing temperatures

|  | Contact angle* | | *p*-value** | |
| --- | --- | --- | --- | --- |
|  | Annealing at 140℃ | Annealing at 60℃ | Annealing at 140℃ | Annealing at 60℃ |
| Cholesterol | 30.6±1.4 | 26.1±0.6 |  |  |
| β-sitosterol | 28.8±3.7 | 31.1±1.4 | 0.488 | 0.013 |
| Ergosterol | 30.0±2.9 | 33.2±3.0 | 0.768 | 0.051 |
| Stigmasterol | 24.9±3.1 | 22.3±1.0 | 0.700 | 0.564 |
| Cholesterol benzoate | 22.4±5.0 | 24.3±4.9 | 0.078 | 0.596 |
| Cholesterol butyrate | 26.9±2.2 | 28.2±4.7 | 0.084 | 0.520 |
| Cholesterol decanoate | 28.5±3.0 | 27.4±2.0 | 0.339 | 0.392 |
| Cholesterol myristate | 35.8±1.4 | 46.1±1.1 | 0.010 | 7.66x10^-5^ |
| Stearyl glycyrrhetinate | 28.8±5.5 | 47.6±5.0 | 0.618 | 0.017 |

*Each measurement was repeated three times (n = 3).

**The *p*‑value represents the significance of the difference compared with the contact angle of cholesterol.


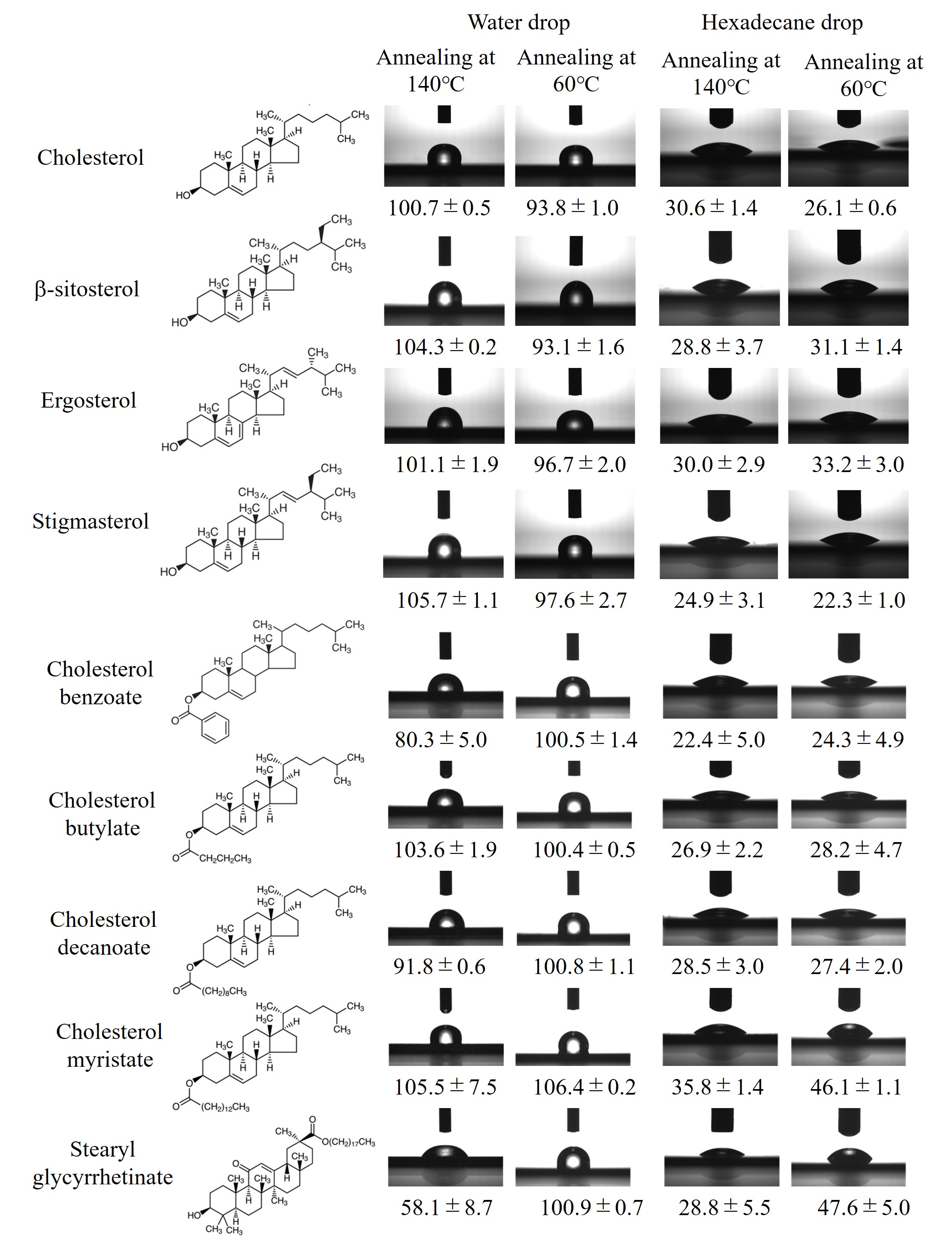


**Supplemental Figure 1.** Contact angle measurements of sterols, as well as cholesterol and its derivatives. The names of each compound and their structures are shown, and actual images of contact angle measurement against a water droplet for estimating hydrophobicity and a hexadecane droplet for determining oleophobicity at two annealing temperatures, together with the contact angle, are presented, similar to Table 1.


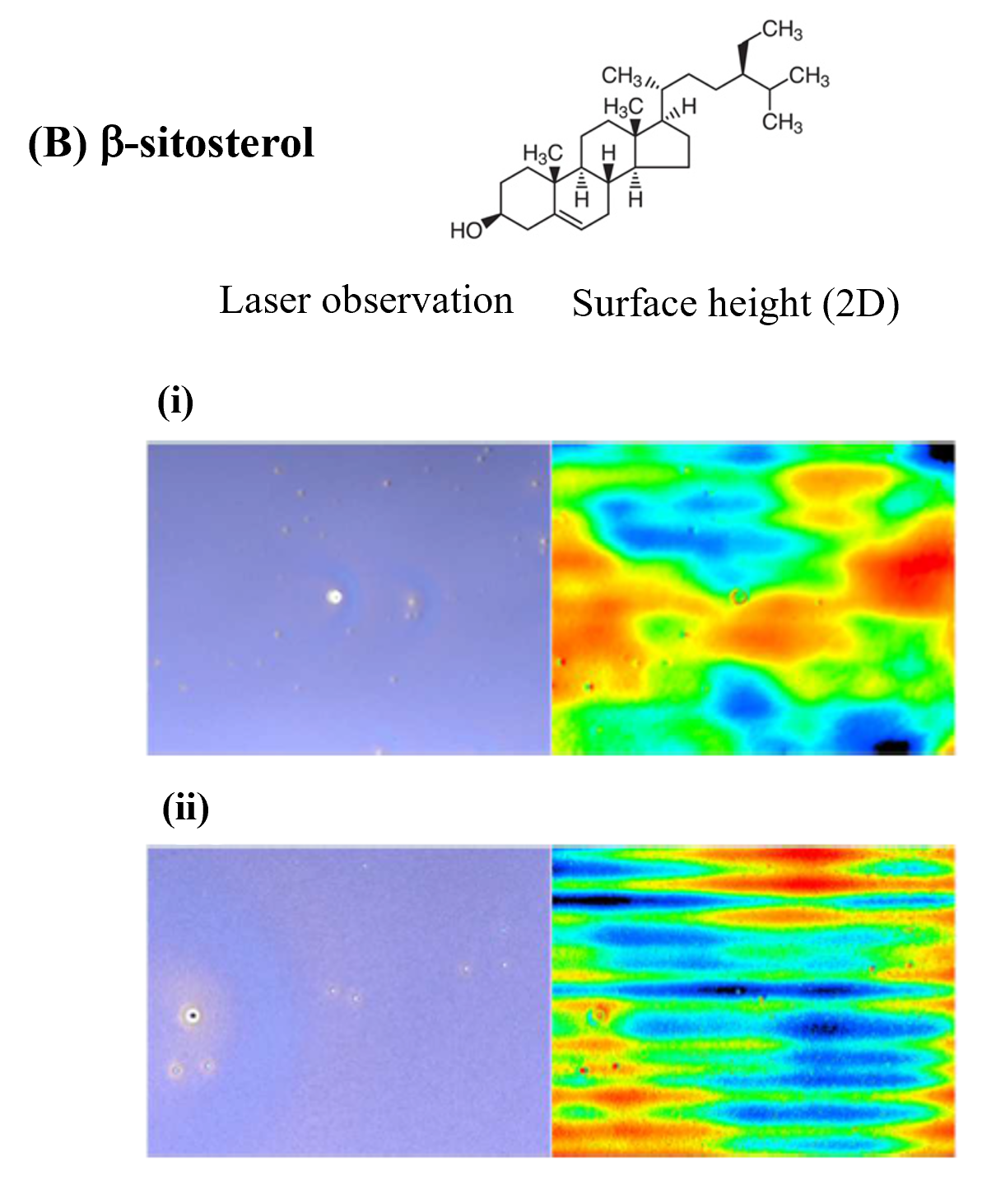

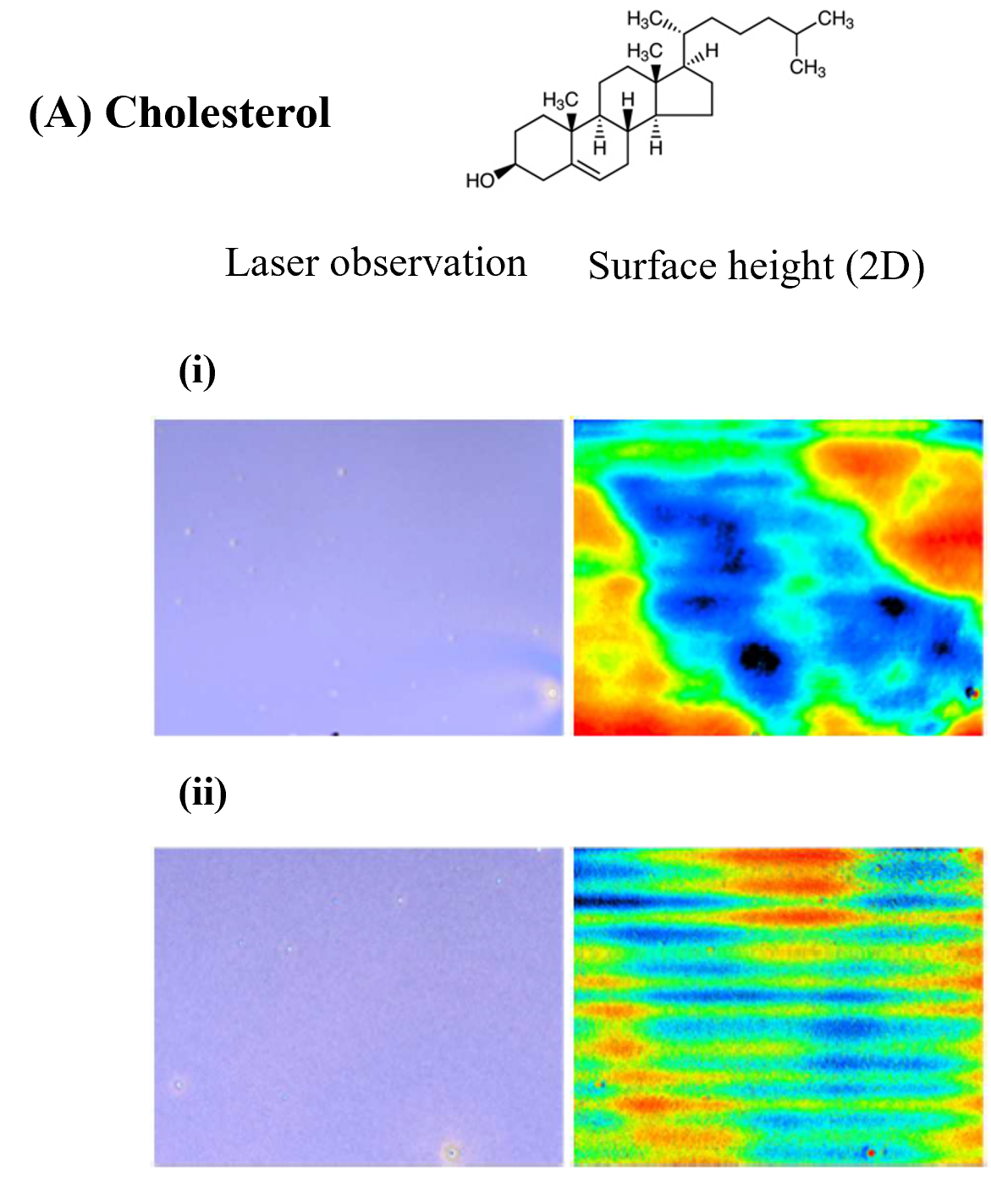


**Supplemental Figure 2.** Laser microscopy observation of silicon wafers coated with (A) cholesterol and (B) β-sitosterol. Their annealing temperature is 140℃. Left panels show laser-based surface observations; right panels represent 2D height maps (red/yellow = higher, blue/green = lower regions). Differences between (i) and (ii) denote the magnifications of the measured locations at 5× and 20×.


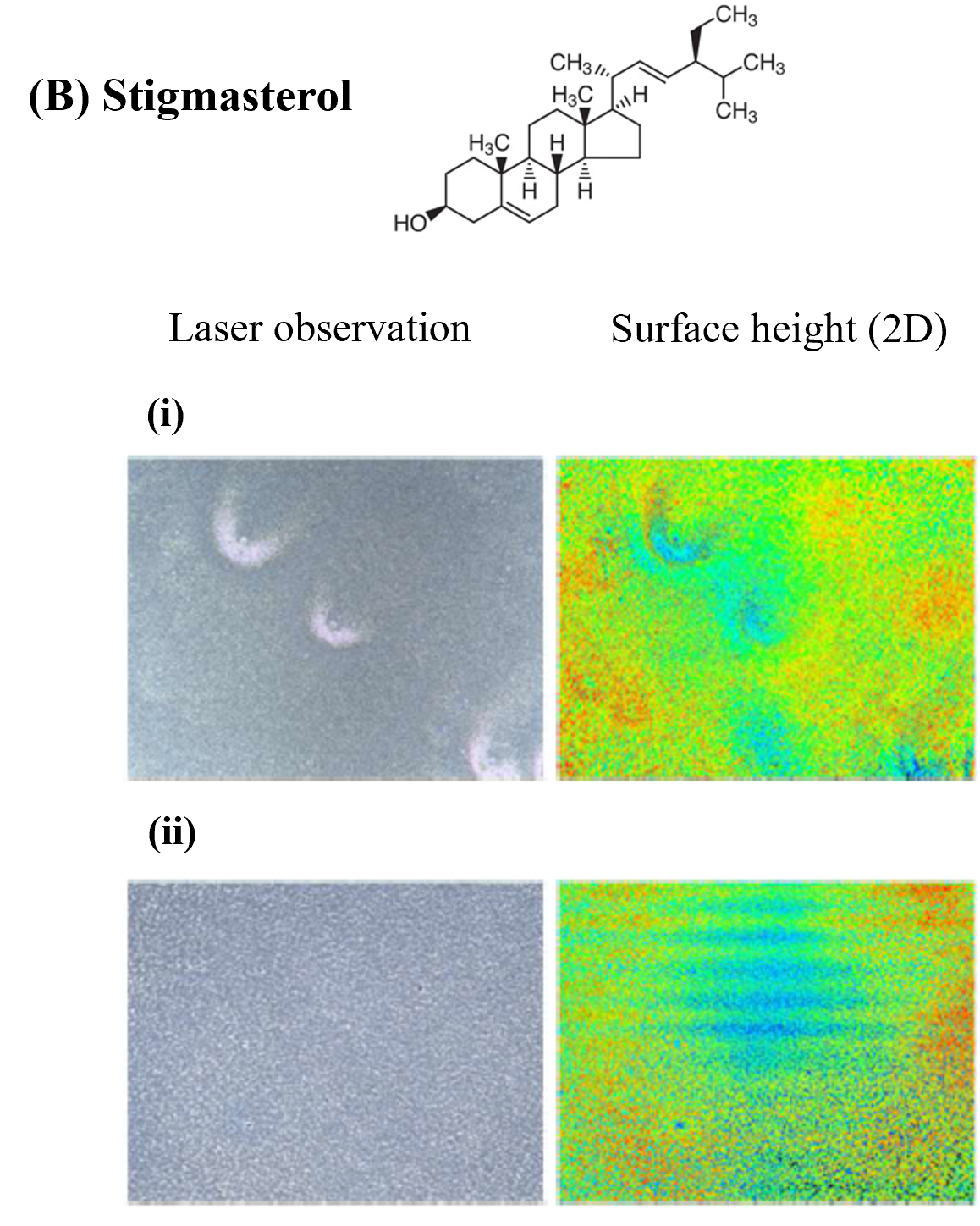

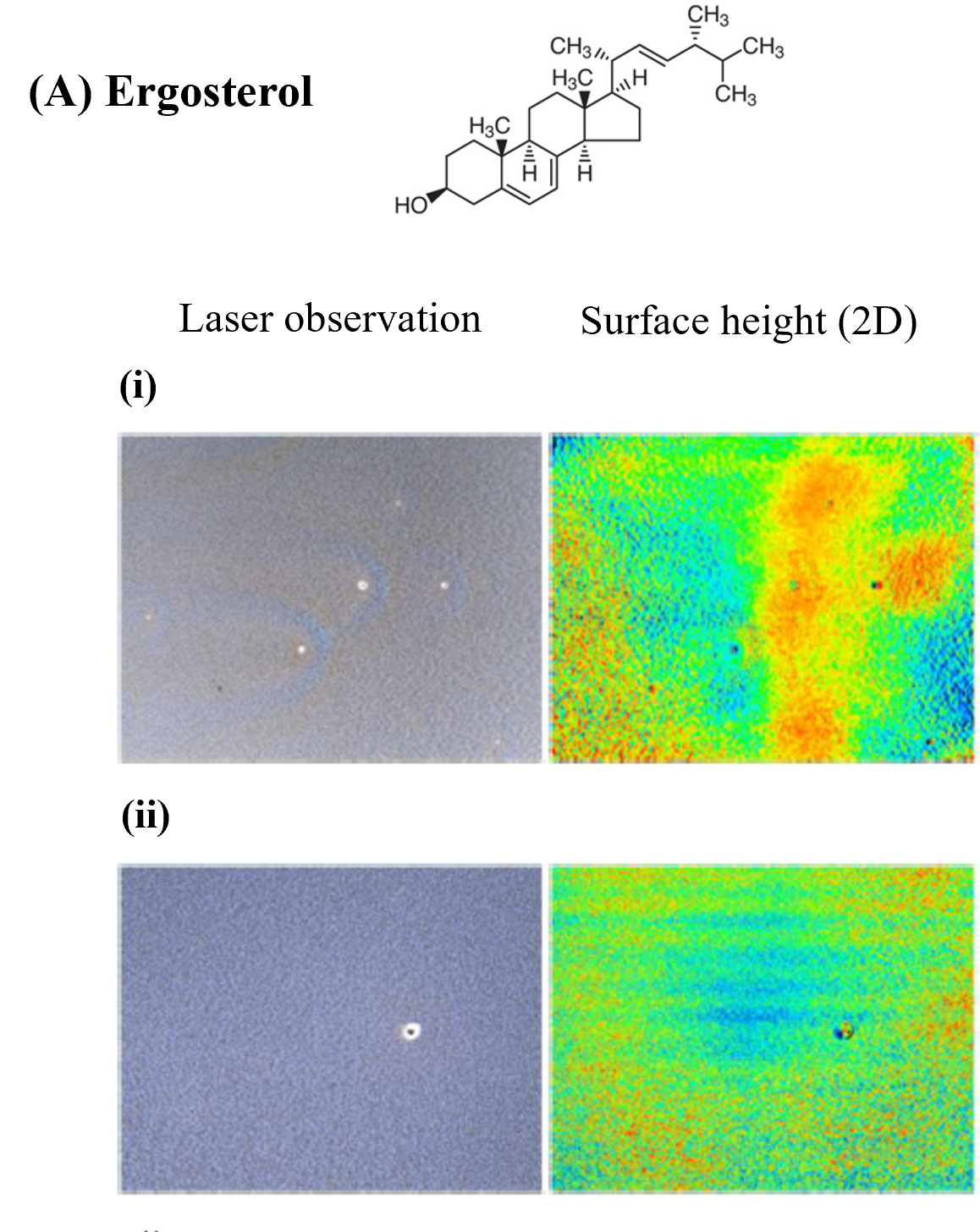


**Supplemental Figure 3.** Laser microscopy observation of silicon wafers coated with (A) ergosterol and (B) stigmasterol. Their annealing temperature is 140℃. Left panels show laser-based surface observations; right panels represent 2D height maps (red/yellow = higher, blue/green = lower regions). Differences between (i) and (ii) denote the magnifications of the measured locations at 5× and 20×.
